# High rates of antibodies against Toscana and Sicilian phleboviruses in common quail *Coturnix coturnix* birds

**DOI:** 10.3389/fmicb.2022.1091908

**Published:** 2023-01-04

**Authors:** Nazli Ayhan, José Domingo Rodríguez-Teijeiro, Marc López-Roig, Dolors Vinyoles, Josep Anton Ferreres, Abir Monastiri, Remi Charrel, Jordi Serra-Cobo

**Affiliations:** ^1^Unité des Virus Emergents (UVE: Aix-Marseille Université, IRD 190, INSERM 1207), Marseille, France; ^2^Departament de Biologia Evolutiva, Ecologia i Ciències Ambientals, Facultat de Biologia, Universitat de Barcelona, Barcelona, Spain; ^3^Institut de Recerca de la Biodiversitat, Universitat de Barcelona, Barcelona, Spain

**Keywords:** phlebovirus, TOSV, SFSV, birds, reservoir, common quail, *Coturnix coturnix*

## Abstract

**Introduction:**

Birds are involved natural cycle of a number of vector-borne viruses in both rural and urban areas. Toscana (TOSV) and Sicilian (SFSV) phleboviruses are sandfly-borne viruses in the genus *Phlebovirus* that can cause diseases in human. However, there is limited information on the role of the birds in sandfly-borne phleboviruses natural cycle and reservoirs ofthese viruses remain unknown.

**Methods:**

In this study, we analyzed Common Quail (*Coturnix coturnix*) sera from Spain to identify the seroprevalence of these two phleboviruses. We tested respectively, 106 and 110 quail serum against TOSV and SFSV from 2018, 2019, and 2021 from two locations in northern Spain with using virus neutralization test.

**Results:**

We identified high neutralizing antibody rates for SFSV (45.45%) and TOSV (42.45%) with yearly fluctuation.

**Discussion:**

This is the first identification of SFSV and TOSV neutralizing antibodies in wild birds. High seroprevalence rates of TOSV and SFSV in quail birds raises the question whether birds have a role as amplifying hosts in the natural cycle of phleboviruses.

## Introduction

Birds play an important role for several arthropod-borne viruses such as *Usutu virus* (USUV) and *Sindbis virus* (SNV) in the old world, and *Saint Louis encephalitis virus* (SLEV), *Eastern Equine encephalitis virus* (EEEV) in the new world, and *West Nile virus* (WNV) worldwide. The transmission cycles of these viruses include arthropod species as vectors and birds as reservoirs. When the vector feeds on infected birds, which are amplifying hosts, they get infected and later on the vector supports virus replication with virions accumulation in the salivary glands to perpetuate transmission to vertebrates (Chan et al., 2015).

*Toscana virus* (TOSV) and *Sandfly Fever Sicilian virus* (SFSV) are both sandfly-borne phleboviruses that belong to the *Phlebovirus* genus in the Phenuiviridae family (order Bunyavirales). Both TOSV and SFSV can infect humans and can cause diseases. SFSV can cause 3-day fever that is characterized by a brutal onset with short but high fever with companion signs such as cutaneous rash headache, photophobia, retro-orbital pain, myalgia and general malaise. TOSV can cause the same manifestations as SFSV, but it can also be responsible for various central and/or peripheral neurological signs, most commonly meningitis and encephalitis (Alkan et al., 2013).

Although antibodies against TOSV and SFSV were found in horses, cats, dogs, cattle, goat and bats (Alwassouf et al., 2016a,b; Ayhan et al., 2017a,^2021^), the amplifying host (if any) remains undiscovered. Since dogs are the reservoir host for certain species of *Leishmania*, it has been hypothesized that they could also play this role for TOSV and/or SFSV. In addition, recent findings have pointed in this direction (Dincer et al., 2015). However, an experimental study conducted with beagle dogs, with two doses of TOSV and SFSV independently, showed absence of clinical manifestation, and viral RNA/infectious virus was not detected in a large variety of clinical samples (blood, saliva, stools, urine, sperm, and bone marrow). Also, the immune response was very limited. The authors conclusions were that dogs are not likely to play a decisive role in the natural transmission and maintenance of TOSV or SFSV (Muñoz et al., 2021). Bats also have been suggested as a reservoir of phleboviruses. However, a study carried out in eight European bat species suggests that bats are not likely to play a major role in the natural cycle of these two viruses (Ayhan et al., 2021).

The knowledge on the role of birds in the ecology and natural cycle of phleboviruses transmitted by sandflies is limited. Recently, TOSV RNA was detected in brain and kidney tissues from three different wild bird species; a greater flamingo (*Phoenicopterus roseus*), a great white pelican (*Pelecanus onocrotalus*), and a black stork (*Ciconia nigra*) (Hacioglu et al., 2017). Interestingly, genetic analysis indicated that RNA sequences belonged to different strains corresponding to each of the two phylogenetic lineages A and B (Hacioglu et al., 2017). In contrast, there is no data supporting an interplay between SFSV and wild birds. Nonetheless, several sandflies species (mostly *Phlebotomus sergenti*) feed on blood of poultry animals (Bongiorno et al., 2003; Svobodová et al., 2003).

In Spain, twelve species of sandflies have been described (Muñoz et al., 2021), including *Phlebotomus perniciosus* and *Phlebotomus papatasi*, which are a proven vector of TOSV (Remoli et al., 2016). SFSV have been identified in *P. papatasi* and *Phlebotomus* ariasi species since now (Izri et al., 2008; Alkan et al., 2013). The presence of the antibodies against TOSV has also been demonstrated in a number of domestic animals (cats, dogs, goats, sheep, cows, pigs, and horses) in Spain (Navarro-Marí et al., 2011). Even, cases of infection due to TOSV were recorded in humans in the country (Eitrem et al., 1991; Mendoza-Montero et al., 1998; Echevarría et al., 2003; Sanbonmatsu-Gámez et al., 2009). In contrast, human cases of infection with SFSV have not been documented in Spain (García San Miguel et al., 2021) and seroprevalence studies reported only a 2.2% rate of positivity (Eitrem et al., 1991).

Migratory birds play an important role in the transmission of diseases due to their great mobility from one area to another, which makes them potential vectors of diseases that can affect domestic animals and human health (Abulreesh et al., 2007). The Common Quail adds to its migratory status the fact of being a game species, which enhances the possible transmission of diseases by direct contact through the food chain. Therefore, the periodic detection of pathogens is of great importance to predict future disease risks for both wildlife and humans (Youssef and Mansour, 2014).

In this study, we investigated if the Common Quail bird could play a role in the natural cycle of TOSV and SFSV. We have collected and analyzed sera from wild quails in Spain to determine the presence of TOSV and SFSV neutralizing antibodies.

## Materials and methods

### Description biological cycle of quail

The common quail is the only migratory Galliformes whose breeding range extends from the Atlantic islands to Lake Baikal and from North Africa to Scandinavia. The European population winters along the Sub-Saharan strip and reaches Europe through three migratory corridors that allow the European metapopulation to be divided, from east to west, into three populations: near-east area (Egypt–Syria route); to a central European area (Tunisia–Italy route); and a western Atlantic area (Morocco–Iberia route). The common quail is a very abundant and widespread Galliformes species. The European population is estimated at 3,320,000—6,720,000 calling or lekking males, which equates to 6,630,000—13,400,000 mature individuals (BirdLife International, 2022). It is found in open habitats including agricultural land where it prefers fields of cereals. It avoids bare soils, trees, and scrub, preferring areas with a dense herb layer less than 1 m tall (Cramp and Simmons, 1980; Aubrais et al., 1986; Tucker and Heath, 1994; McGowan et al., 2013). Most common quails are migratory individuals. In autumn, they leave Europe and go to the Sub-Saharan region of Africa where they overwinter (Rodríguez-Teijeiro et al., 2012). It is a game species that can be hunted in eight European countries (Austria, Bulgaria, Cyprus, France, Greece, Italy, Malta, Portugal, and Spain) (Perennou, 2009).

### Data and sample collection

**FIGURE 1:**
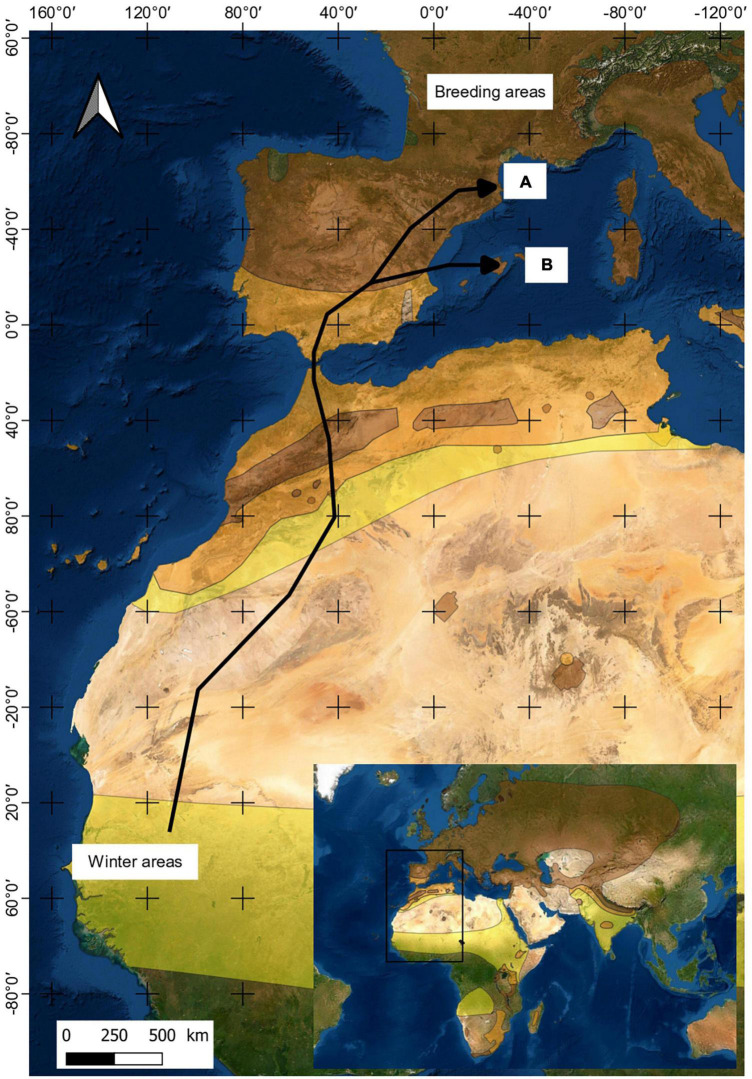
Map showing one of the possible migration routes of common quails (*Coturnix coturnix*) from winter areas to breeding areas. Letters correspond to Alp **(A)** and Mallorca **(B)** locations.

One hundred and eleven male common quails were collected from two Spanish locations in 2018, 2019, and 2021: croplands near Alp, a village located in the Pyrenees of Catalonia, and croplands located in the middle of Mallorca Island, near Sineu and Vilafranca de Bonany villages (Figure 1). Quails were captured by spreading a 10 × 12 m net horizontally over the cereal plots during the reproduction period (Rodríguez–Teijeiro et al., 2010). Two days were chosen in the middle phase of their stay in the breeding area (April–July both inclusive), and the maximum number of males in the study area were captured. We distinguish two age categories for all quails: juveniles, born in the breeding season, and adults born in the previous year’s breeding season. All individuals were ringed. Blood samples were obtained by a small puncture made in the proximal jugular vein. Samples were stored at 4°C for a few hours before undergoing 12,100 *g* centrifugation for 20 min. Serum and clot pellets were separated, and sera were stored at −80°C until further processing.

### Sero-neutralization assay

Quail sera were inactivated at 56°C for 30 min. After inactivation, 15 μL of sera were diluted from 1/10 to 1/80 and mixed at a 1:1 ratio with 100TCID_50_ viral suspension of TOSV (strain MRS2010–4319501) and SFSV (strain Sabin) in 96-well microliter plates in parallel. After 1 h incubation at 37°C, a 100 μL suspension of Vero cells (ATCC CCL-81) containing approximately 2× 10^5^ cells/mL was added to each well providing two-fold final dilutions between 1:20 and 1:160 (Alwassouf et al., 2016a). Negative controls containing minimum essential medium (MEM), with or without serum, together with positive controls were included in each microplate. After 5 days (for TOSV) and 6 days (for SFSV), the microplates were read under an inverted microscope, and the presence (neutralization titer at 20, 40, 80, and 160) or absence (no neutralization) of the cytopathic effect was noted. Cutoff value for positivity was set at titer ≥40 as in previous studies (Alwassouf et al., 2016b; Ayhan et al., 2021).

### Statistical analysis

All variables (described in Table 1) were screened using a logistic regression univariate analysis and a chi-square test to check for statistically significant associations with TOSV- and SFSV-positive serological status. Odds ratios and 95% confidence intervals (Cis) were used to assess the strength of association between risk factors and TOSV and SFSV serostatus. In all tests, significance was set at *p*-value < 0.05. Data were analyzed using R software version 2.8.1 (R Core Team, 2022).

**TABLE 1:** Description of risk factors for positive sera of *Toscana virus* (TOSV) and *Sandfly Fever Sicilian virus* (SFSV) included in univariate analysis. Data were collected from 111 *Coturnix coturnix* sampled in 2018, 2019, and 2021.

Variable	No. of animals
**Location**	
Alp	95
Mallorca	16
Total	111
Year	
2018	19
2019	55
2021	37
Total	111
**Age class**	
Juveniles	30
Adults	79
Undetermined	2
Total	111

## Results

### Quail sera collection

Respectively, 19, 55, and 37 quail sera were collected in 2018, 2019, and 2021 from the Alp and Mallorca regions in Spain. The Mallorca region was only sampled in 2019, and we obtained 16 samples. Some sera did not have sufficient volume to test for both viruses, and for this reason 110 and 106 were used to test for SFSV and TOSV, respectively. No quail was recaptured during the period of study.

### Serological analysis

**FIGURE 2:**
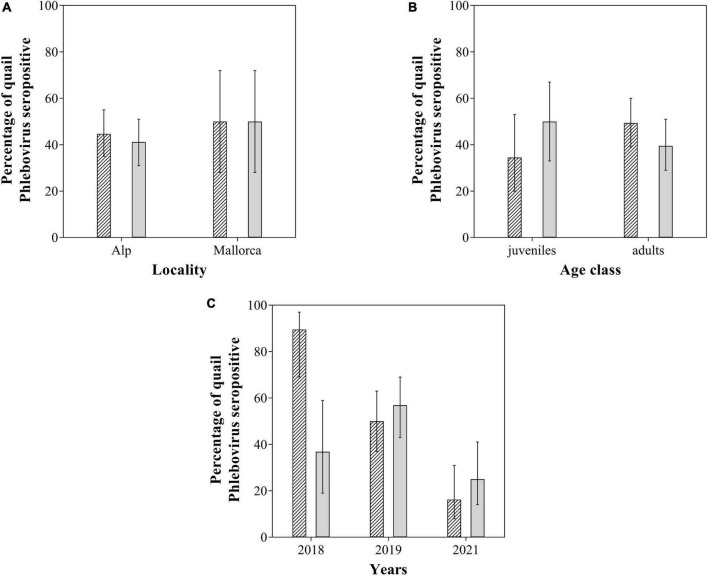
*Sandfly Fever Sicilian virus* (SFSV) (scratched) and *Toscana virus* (TOSV) (uniform gray) seroprevalence as a function of **(A)** location, **(B)** age classes, and **(C)** years. Error bars represent 95% binomial confidence intervals (CIs).

We investigated the presence of SFSV antibodies, and a total of 110 sera were obtained, of which 50 (45.45%; 95% CI: 36.46–54.76) were seropositive (Supplementary Table 1). We tested 106 sera against TOSV, of which 45 (42.45%; 95% CI: 33.47–51.96) were seropositive (Supplementary Table 1). Twenty-nine samples were seropositive for both viruses (27.62%). Although the level of sampling was different between the two locations, seroprevalence of SFSV and TOSV did not vary significantly between locations. Also no significant differences of seroprevalence for either virus were found between age classes (Table 2). A significantly higher seroprevalence of SFSV was observed in 2018 compared to 2019 and 2021. The seroprevalence of TOSV was significantly higher in 2019 compared to 2021 (Table 2 and Figure 2).

**TABLE 2:** Univariate analysis of risk factors for positive sera of *Sandfly Fever Sicilian virus* (SFSV) and *Toscana virus* (TOSV): Proportion seropositive, odds ratios with 95% confidence intervals (CIs) and chi-square *p*-values for variables included in the analysis.

SFSV						
Variable	Category	*n*	Seropositive proportion	Odds ratio	95% CI	Chi-square *P*-value
**Location**						
	Alp	94	0.447	1.000		
	Mallorca	16	0.500	1.238	0.428–3.577	0.693^a^
**Year**						
	2018	19	0.895	1.000		
	2019	54	0.500	0.118	0.024–0.559	0.007
	2021	37	0.162	0.023	0.004–0.125	<0.001
**Age class**						
	Juveniles	29	0.345	1.000		
	Adults	79	0.494	1.852	0.766–4.483	0.171
TOSV						
Variable	Category	*n*	Seropositive proportion	Odds ratio	95% CI	Chi-square *P*-value
**Location**						
	Alp	90	0.411	1.000		
	Mallorca	16	0.500	1.432	0.493–4.160	0.509^a^
**Year**						
	2018	19	0.368	1.000		
	2019	51	0.569	2.300	0.764–6.684	0.141
	2021	36	0.250	0.571	0.172–1.896	0.360^b^
**Age class**						
	Juveniles	28	0.500	1.000		
	Adults	76	0.395	0.652	0.273–1.560	0.337

a: comparison between location (same year 2019) *p*-value = 0.504.

b: comparison between years (2019–2021) *p*-value = 0.011.

## Discussion

The “Global Vector Control Response (GVCR) 2017–2030” was approved by the World Health Assembly in 2017 and provided strategic guidance to countries for urgent strengthening of vector control to prevent diseases and outbreaks (World Health Organization [WHO], 2022). However, phleboviruses transmitted to humans and animals by sandflies are relatively neglected and are not on the priority list of national and international public health agencies. Even though the problem is neglected, the public health impact of sandfly-borne infections is relevant in countries along the Mediterranean and expanding northwards. The problem is developing in Europe because, due to climate and environmental changes and human activity, phlebovirus vectors have increased in frequency (Maroli et al., 2013; Alten et al., 2016). The niche modeling approach predicts climatically suitable areas for sandflies as being not only in the Mediterranean region but also in Central and Northern Europe, e.g., countries like Austria, Switzerland, and Germany, and even regions in Scandinavia and the mainland of Great Britain (Koch et al., 2017). On the other hand, the great mobility of people and trade also favors the dispersion of sandflies. Considering the growing expansion of phlebovirus vectors and the millions of people that are potentially exposed, it is important to find phlebovirus reservoirs and to understand their cycles in order to propose preventative measures. Our study aims to contribute to knowledge on the circulation of SFSV and TOSV in animals that have so far been studied infrequently.

Several studies demonstrated the circulation of SFSV in a wide geographical area, including Asia, south Europe, and Africa; in contrast TOSV circulation is restricted within the Mediterranean basin (Ayhan et al., 2017b). If the maintenance and the transmission of these viruses appear highly dependent on the abundance and the distribution of the relevant sandfly species, there still are in our knowledge of rural and/or urban cycles because to date, the vertebrate reservoir host for TOSV and SFSV, if any, remains undiscovered.

In this study, we found for the first time SFSV and TOSV neutralizing antibodies in wild birds. Our results show rates of neutralizing antibodies that were among the highest ever detected in vertebrates (Alwassouf et al., 2016b; Ayhan et al., 2021). Moreover, if the cut-off is decreased and moved from 40 to 20, NT Abs against SFSV and TOSV would be observed in 59% and 58.5% of quails, respectively. These results contrast with the unbalanced NT Abs rates previously observed in dogs from Portugal, Cyprus, Crete, and mainland Greece (Alwassouf et al., 2016b). Interestingly, the same quail serum often contained NT Abs against TOSV and SFSV. On the other hand, we found significant interannual differences in seroprevalence in quails in concordance with the dynamics of viruses in reservoir species (Serra-Cobo and López-Roig, 2017; Table 2 and Figure 2). We also found NT Abs against TOSV and SFSV in some common swift (*Apus apus*) from Spain (unpublished data). Although SFSV RNA and TOSV RNA have been rarely detected in vertebrates, TOSV RNA has only been recently detected for the first time in birds. Taking into account our results and that birds can be reservoir hosts for other zoonotic arboviruses, we can say that they could be also reservoirs of the natural cycle of SFSV and TOSV.

**FIGURE 3:**
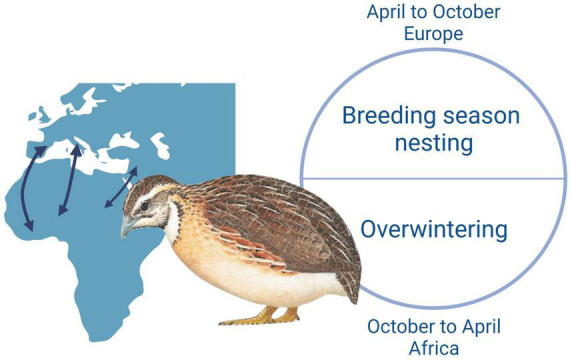
The biological cycle and migration routes of quails. Created with BioRender.com.

The quails analyzed are migratory birds who come from Maghreb or Sub-Saharan Africa into Europe in April to breed. In October, they leave Europe for Africa where they overwinter (Figure 3; Perennou, 2009). Accordingly, they stay in the Mediterranean area at least from April to October, which is the period of activity of the sandfly. Entomological surveys in several countries of the Mediterranean basin showed the presence of two sandfly species: *P. perniciosus* and *P. papatasi*, which are the known vectors of TOSV and SFSV, respectively. The presence and abundance of *P. perniciosus* were shown on the island of Mallorca and the region close to the Pyrenees of Catalonia (Alcover et al., 2014; Gálvez et al., 2020). However, the low seroprevalence described in vertebrates tested for the presence of antibodies against SFSV suggests that this virus might circulate at very low levels in European regions where quails are breeding. In addition, during the last decade, SFSV-like viruses were detected by molecular methods in sandflies collected in Tunisia (Utique virus and Saddaguia virus) (Fares et al., 2015; Dachraoui et al., 2016); accordingly, it is plausible that quails get infected by SFSV or SFS-like viruses. When we consider the known distribution of SFSV in Africa and low seroprevalence of SFSV in Spain, we can speculate Africa as a probable infection region. Entomological surveys introduced two predominance sandfly species: *P. papatasi* and *P. perniciosus* in Algeria and Morocco, of the genus *Phlebotomus*, from which *P. papatasi* is a known vector of SFSV (Boudrissa et al., 2012; Boussaa et al., 2016; Muñoz et al., 2021). In addition, SFSV was detected from *P. ariasi* in field-collected sandflies in Algeria, indicating the virus circulation in North Africa (Izri et al., 2008). The wide distribution of these sandfly species indicate that both viruses circulate in the Mediterranean region and that common quails are likely to become infected on the two shores of the Mediterranean.

Several studies have shown that bird species can be a potential blood source for a number of sandfly species, since they display the lowest defensive behavior compared to other animals (Svobodová et al., 2003). In this sense, it is likely that when different types of vertebrate hosts are available, sandflies tend to choose poultry over mammals. This behavior has been observed repeatedly in rural areas of Maghreb where poultry, donkey, goats, and sheep are sheltered together.

Taking into account the results obtained and the recent detection of TOSV in large migrating birds (Hacioglu et al., 2017), it should be considered that quails (and possibly other birds) can be a reservoir or amplifying hosts for TOSV and SFSV, and possibly other sandfly-borne phleboviruses. Quails are considerably more mobile than terrestrial mammals and can spread pathogens over long distances. In this sense, they are possibly good reservoirs and have the potential to rapidly and widely spread viruses. Within the European Union, at least 1,607,964 quail are shot each year, which is equivalent to 40% of the average breeding population of the EU countries (Hirschfeld et al., 2019). This adds a degree of concern regarding the risk of spreading viruses, since, during hunting activities, both dogs and humans are in direct contact with quail.

Our results, together with previous data, constitute a body of evidence that shows that the common quail plays a prominent role in the ecology of these viruses. Future research should address this hypothesis using both experimental studies and wildlife based studies.

## Data availability statement

The original contributions presented in this study are included in the article/Supplementary material, further inquiries can be directed to the corresponding authors.

## Ethics statement

The animal study was reviewed and approved by the ethical recommendations of the European Union and Spanish Legislation (Spanish Law 5/1995 and Decree 214/1997) and was approved by the Ethics Committee on Animal Experimentation from the University of Barcelona.

## Author contributions

NA, RC, and JS-C conceived the study. NA, JR-T, ML-R, DV, JF, and AM developed the methodology and analyzed and interpreted the data. NA performed serological assays and wrote the manuscript. JR-T, ML-R, DV, JF, and AM designed, conducted, and provided the bird serum samples the clinical samples. All authors edited successive drafts and approved the final version.
