# Supplementary material for: High rates of antibodies against Toscana and Sicilian phleboviruses in common quail Coturnix coturnix birds
Source: Front Microbiol. 2023 Jan 4;13:1091908. doi: 10.3389/fmicb.2022.1091908 (PMC9846092; doi:10.3389/fmicb.2022.1091908)
Supplement: Supplementary file 1 [file Table_1.DOCX]

Table S1

| **SFSV** | **2018** | | **2019** | | **2021** | | **Total** | |
| --- | --- | --- | --- | --- | --- | --- | --- | --- |
|  | n | % | n | % | n | % | n | % |
| Nº of samples | 19 |  | 54† |  | 37 |  | 110 |  |
| Positive | 17 | 89.5 | 27 | 50.0 | 6 | 16.2 | 50 | 45.5 |
| Negative | 2 | 10.5 | 27 | 50.0 | 31 | 83.8 | 60 | 54.5 |
| 0 | 2 | 10.5 | 16 | 29.6 | 27 | 73.0 | 45 | 40.9 |
| 1/20 | 0 | 0.0 | 11 | 20.4 | 4 | 10.8 | 15 | 13.6 |
| 1/40 | 11 | 57.9 | 5 | 9.3 | 4 | 10.8 | 20 | 18.2 |
| 1/80 | 6 | 31.6 | 5 | 9.3 | 2 | 5.4 | 13 | 11.8 |
| ≥1/160 | 0 | 0.0 | 17 | 31.5 | 0 | 0.0 | 17 | 15.5 |

† One sample of sera from 2019 was not analyzed.

|  |  |  |  |  |  |  |  |  |
| --- | --- | --- | --- | --- | --- | --- | --- | --- |
| **TOSV** | **2018** | | **2019** | | **2021** | | **Total** | |
|  | n | % | n | % | n | % | n | % |
| Nº of samples | 19 |  | 51† |  | 36† |  | 106 |  |
| Positive | 7 | 36.8 | 29 | 56.9 | 9 | 25.0 | 45 | 42.5 |
| Negative | 12 | 63.2 | 22 | 43.1 | 27 | 75.0 | 61 | 57.5 |
| 0 | 12 | 63.2 | 10 | 19.6 | 22 | 61.1 | 44 | 41.6 |
| 1/20 | 0 | 0.0 | 12 | 23.5 | 5 | 13.9 | 17 | 16.0 |
| 1/40 | 6 | 31.6 | 13 | 25.5 | 7 | 19.4 | 26 | 24.5 |
| 1/80 | 0 | 0.0 | 5 | 9.8 | 2 | 5.6 | 7 | 6.6 |
| ≥1/160 | 1 | 5.3 | 11 | 21.6 | 0 | 0.0 | 12 | 11.3 |

† Four and one sera samples from 2019 and 2021 respectively was not analyzed.
